# The Circular Experimentation Workbench – a Lean and Effectual Process

**DOI:** 10.1007/s43615-022-00239-w

**Published:** 2022-12-07

**Authors:** Nancy Bocken, Matthew Coffay

**Affiliations:** 1 School of Business and Economics, Maastricht Sustainability Institute, Maastricht University, Tapijn 11 Building D, P.O. Box 616, 6200 MD Maastricht, The Netherlands; 2 Department of Business Administration, Webstern Norway University of Applied Sciences, Inndalsveien 28, 5063 Bergen, Norway

**Keywords:** Circular business models, Circular economy, Sustainability, Effectuation, Lean startup, Business experimentation

## Abstract

Circular economy has become an important goal for companies to address climate change and pressing resource issues. Yet, the process of circular business experimentation is highly uncertain. While the lean startup has been applied to the circular experimentation process, the concept of effectuation has only been used to a limited extent, despite its potential. We investigate the following question: To what extent can lean startup and effectual thinking be combined to support the circular business model innovation process? We conducted 10 workshops where we combined these concepts with circular economy thinking. A novel process – the Circular Experimentation Workbench – was developed and evaluated to inspire participants to start experimenting with the circular economy. We found that lean startup and effectuation principles are highly complementary. Effectual questions can support the development of successful circular experiments. Our results were potentially limited by constraints related to the workshop format and action research method. Future research could build on the complementary perspectives of lean startup and effectuation to help accelerate the circular economy transition.

**Supplementary Information:**

The online version contains supplementary material available at 10.1007/s43615-022-00239-w.

## Introduction

The circular economy is seen as an important avenue to combat global challenges such as climate change, resource scarcity, waste, and biodiversity issues. The promise is that the circular economy can create various win–win situations on an individual, business, and macro scale, such as the reduction of resource use, competitiveness, new revenues and cost reductions, and job creation [1, 2]. Circular economy should not be seen as a threat but rather as an opportunity as in particular younger consumers give increasing preference to sustainable products and circularity allows companies to make better use of existing products and resources [3, 4]. The resource-conserving strategies of a circular economy can be classified according to strategies for narrowing resource loops (i.e., using fewer resources per product), slowing resource loops (i.e., using products for longer), closing resource loops (i.e., recycling), and regenerating resources (i.e., using renewable resources and regenerating the natural environment) [5–7].

Scholars have noted the great interest of business and policy makers in circular economy [8] and new business models [9–11]. This is perhaps not surprising given the potential of sustainable and circular business models to generate significant sustainability impacts thanks to their holistic lens on how business is done and the incorporation of various stakeholders, including the natural society and environment, in the company purpose, vision, and performance indicators [12, 13]. Furthermore, in addition to the potential for substantial sustainability impacts, there is a growing awareness in conventional business circles of the financial upside and value-creation potential of circular and sustainable business models [4]. Examples of circular business models include IKEA’s buyback and resell service designed to increase the lifetime of furniture and slow the loop, MUD Jeans’ lease a jeans concept to close the loop, and Patagonia’s regenerative organic agriculture model to regenerate the natural environment [14]. Furthermore, “gap-exploiters” pursue circular business models where the existing industry is lagging behind in pursuing circular business opportunities in sectors like ICT and electric vehicle batteries [15, 16]. In general, more radial circular business models, for instance, around slowing loops, have only been implemented to a limited extent in large businesses [17, 18]. At the same time, there are many circular startups [19], but it takes time before startups reach scale and scale impact, and there is a large failure rate.

This paper focuses on the business transition toward a circular economy, specifically by companies pursuing circular business models, and the role of tools and methods in it. Popular tools in business include the lean startup [20, 21], which takes an iterative approach of building, measuring, and learning about business models through experimentation based on hypotheses about the future business and testing ideas with customers early on. The lean startup was developed originally for startups [21] but is now widely used by large businesses [22], also in a sustainability context [23, 24]. Effectuation is an entrepreneurial approach based on leveraging the resources available [25]. Entrepreneurs leverage who they are (traits, abilities), what they know (expertise, experience), and whom they know (social and professional networks). Using these means, the entrepreneurs begin to imagine and implement possible effects that can be created with them [25]. In contrast to lean startups, effectuation has not been widely used in existing businesses. However, the effectuation focus on using “what is available” can be highly valuable in organizations that have to balance between continuing their existing business model which they are vested in and have allocated most resources to, with the new business model being tested.

This research seeks to understand to what extent notions from the lean startup and effectuation may be bridged to support businesses in their transition toward the circular economy. Furthermore, circular and sustainable business model tools’ reviews have highlighted the need for tools supporting the process of experimenting and piloting, as well as transforming the organization for the circular economy [26, 27]. The following question is investigated: *To what extent can lean startup and effectual thinking be combined to support the circular business model innovation process?*

The next section describes the background of circular business model experimentation, effectuation and lean startup thinking, and the research focus in more detail. The “Method” section describes the action-oriented design science method to develop and test the novel Circular Experimentation Workbench process. The “Discussion” section reflects on the complementarities and challenges when using lean startup and effectuation to support the circular business model experimentation process. Finally, the “Conclusions” section summarizes the contributions and next steps.

## Background

This study seeks to bridge the research areas of circular business model experimentation, concepts on theories on effectuation and lean startup, and tools and methods. The following briefly reviews relevant literature to illuminate the research gap.

### Circular Business Model Experimentation

Circular business models seek to create positive value for the environment, society, and customer [28], through strategies such as narrowing the loop (efficiencies, using less), closing the loop (recycling), slowing the loop (durability, product life extension), and regeneration (improving the natural and social environment) [5, 14, 29]. Sustainable and circular business models are important in the context of the circular economy because they have the potential to take a holistic view of the way business is done [12]. Circular business models are not only about the products but also in the way products and services get delivered to the customer so that the total environmental impact of these can be significantly reduced through efficiencies in production, use, and reuse phases [30]. In this way, companies might be able to achieve their ambitious circular economy goal more quickly.

Yet, circular business models do not emerge automatically and are only still emerging in practice [18, 31]. On the contrary, they need to compete with dominant existing linear business models, so significant experimentation is required to test the desirability, feasibility, viability, and sustainability of such new business models in practice [32]. For example, the case of experimentation with a circular business model in the fast-moving consumer goods industry, trialing a refill model, illuminated the need for convenience and accessibility as well as affordability, and a clear demonstration of the environmental improvement of such a model to the customer for successful adoption [33]. Experimentation is not only about the learning process but also about strategic legitimation, in particular in existing businesses [34]. Experimentation is becoming a more important theme in circular economy literature [27, 35].

Circular business model experimentation may be described as follows. It is *“an iterative approach to develop and test circular value propositions in a real-life context with customers and stakeholders, starting with a shared goal. It involves rapid learning based on empirical data to provide evidence on the viability of circular value propositions. Iterations involve increased complexity of experiments. There is a learning focus on initiating wider transitions, such as transforming consumer behaviours for the circular economy.”* [36]

Companies are indeed experimenting with new circular business models in practice. Examples include rental, subscription, and lease to slow and close resource loops. Service-oriented business models can achieve a factor of 2 to 10 improvement in environmental impact reduction compared to just selling a product when the model is set up in the right way [37]. Companies are starting to launch several circular business models in different countries [38]. Experiments are necessary to understand first whether a business model is desirable, feasible, viable, and sustainable [32]. Second, it allows companies to test to what extent the business model “works” or needs to be adapted in different contexts [38]. For example, the geographical landscape and infrastructure might determine the success of a bike-sharing model, or the regulatory environment might provide certain boundary conditions for a new circular business model that reuses materials [38].

The problems are that experimentation with circular business models is insufficiently happening routinely in practice. Moreover, only a small number of tools support the experimentation and piloting phase, as identified in a review by Pieroni et al. [27].

### Effectuation and Lean Startup Type of Experimentation

Startups can be seen as one big experiment to test whether a business model works in practice [20, 21]. It is perhaps not surprising that tools such as lean startup, originating from startup literature, are being used by incumbent businesses and, notably, also large incumbents [22, 23].

Lean startup is an iterative approach to test hypotheses about a future business in a relatively short, time-bound, and cost-effective manner [21]. It contains iterative “build-measure-learn” cycles. A minimum viable product (MVP) is typically built as an experiment before committing too many resources to a full prototype [39]. One example is the “Wizard of Oz” simulation [39], where people manually, rather than technology, deliver the service provided. Think, for instance, about a new delivery system where all facets are still all operated manually to test whether people would use it before building it in full. These low-cost, low-resource characteristics also fit a corporate environment where most resources are allocated toward sustaining the existing business model and it is a challenge to gradually transform toward a more sustainable or circular business model [24, 40]. At the same time, recent scholarship highlights the need for lean startup methods to be adapted to work in incumbent contexts [41].

Perhaps surprisingly, another popular startup theory – effectuation – developed by Sarasvathy [25], is used less in the corporate sphere despite its potential. Sarasvathy developed the following principles for entrepreneurs: (1) bird-in-the-hand (use available means, make do with what you have), (2) affordable loss (what can I accept to lose), (3) crazy quilt (stakeholder commitments expand means and shape the enterprise), (4) lemonade (leverage uncertainty and exploit unexpected opportunities), and (5) the pilot-in-the-plane (actor agency shapes the future) [25]. These principles can support entrepreneurs in developing and shaping their ventures. However, principles such as focusing on using available means and the crazy quilt (working with familiar stakeholders) would fit a corporate context as both would reduce (search) cost. The lemonade and pilot-in-the-plane principles [25] might provide additional inspiration to help shape a new corporate context in an uncertain environment [42]. Some studies have investigated SMEs in relation to effectuation. Evald and Senderovitz [43] found effectuation to be useful for SMEs to be more innovative. Uzhegova and Torkkeli [44] found that effectual logic in SMEs can lead to more responsible business practices. More generally, Brettel et al. [45] find that effectuation is positively linked to success in highly innovative contexts, and Futterer et al. [46] found that effectuation can be most beneficial in a high-growth corporate context.

As contrasted with causal thinking (scientific approach of hypothesis testing fitting lean startup), effectuated approaches to innovation involve the leveraging of available means to create opportunities. Traditional causal approaches to innovation can be likened to preparing a meal with a recipe: The recipe is selected first, ingredients are purchased, cooking implements are acquired, and a meal is prepared [25]. By contrast, effectuation implies seeing what is in the kitchen and improvising “one of many possible desirable meals” [25, p. 245]. In effectuation theory, entrepreneurs start with the following: (1) Who they are – their traits, tastes, and abilities; (2) What they know – their education, training, expertise, and experience; and (3) Whom they know – their social and professional networks. Using these means, the entrepreneurs begin to imagine and implement possible effects that can be created with them [25]. Effectual entrepreneurs transform market failures into sustainable solutions by self-selecting stakeholders [47].

Given the vast knowledge, technological, and capital resources available in incumbent firm environments — combined with what are often well-developed networks for collaboration — effectuated thinking promises to help firms leverage existing strengths and resources to develop new value propositions, innovate their business model, and actively shape and create market opportunity. Though this potential was recognized more than a decade ago, with Chesbrough [48], p. 362] suggesting companies “must adopt an effectual attitude toward business model experimentation,” there is still a considerable practice gap. Some studies have investigated the benefits of effectual thinking, mainly by retrospectively analyzing R&D projects or ventures [45, 46]. However, few, if any, have analyzed such processes “in action.” Exceptions include, e.g., the work by Keskin et al. [49], who followed new ventures over a longer period and found both effectual and causal processes to be at play, and Brown et al. [50], who used effectual notions in a workshop setting. Moreover, and importantly for the context explored here, scholars have not reached an agreement on whether (and if yes, how) these theories can be reconciled.

Critically, the inventor of effectual theory, Sarasvathy [51], has noted that scholars have reduced effectual action to the bird-in-hand principle without discussing either the crazy quilt (stakeholder self-selection) or the pilot-in-the-plane (co-creation) principles, or worse still, equating effectuation to experimentation. She emphasizes that “effectuation is not experimentation” [51, p. 7–8]. She argues that the scientific method of hypothesizing present in lean startup is helpful only with regard to predictable aspects of reality. As entrepreneurship deals with the unpredictable and the fundamentally unknowable, seeking to validate or falsify claims is not a useful strategy and definitely not the only or most suitable strategy available [51]. There are more fundamental differences: While lean startup type of causal reasoning focuses on expected returns, effectual reasoning emphasizes affordable loss [25]. Lean startup is about understanding fit compared to the competition, while effectual reasoning is built upon strategic partnerships; and while lean startup leverages pre-existing knowledge and prediction, effectual reasoning stresses the leveraging of contingencies [25].

According to Sarasvathy [52, p. 9], entrepreneurs often think effectually: “*They believe in a yet-to-be-made future that can substantially be shaped by human action; and they realize that to the extent that this human action can control the future, they need not expend energies trying to predict it*.” Moreover, Sarasvathy [52] argues that rather than contemplating the extent to which the future is shaped by human action, it is not much use trying to predict it. Rather, it is much more useful to understand and collaborate with people who are engaged in the decisions and actions that influence the future (see, e.g., [13]). This is especially relevant in sustainability and circularity contexts, where the wicked nature of sustainability challenges implies considerable uncertainty [53]. Uncertainty in entrepreneurial contexts, however, can be overcome not by just gathering the correct information about the external environment but by participating in the process of gradually transforming it [54, 55].

However, businesses also need to understand where they fit against the competition and how they make an attractive offering by iterating their proposition, as done in the lean startup approach [21]. This potentially makes the combination of both approaches strong. Furthermore, there are many synergies between the approaches like the iterative approach, early stakeholder-involved learning, and low-resource approach of the method.

Table 1 highlights some of the differences and similarities between lean startup and effectuation.

**Table 1:** The lean startup vs. the effectual approach. (Source: building on [21, 25, 47, 51])

	Lean startup	Effectuation
Premises	Iterative build-measure-learn cycles	Start with available means: who you are, what you know, who you know
Focus	Expected return	Affordable loss
Competition vs. collaboration	Understanding competitive positioning	Forging strategic partnerships
Method	Scientific method	Entrepreneurial method
Approach	Scientific approach (as if) Knowledge and prediction Test hypotheses, e.g., A-B split testing “Value-neutral”	Effectual approach (even if) Leveraging contingencies Co-create hypotheses “worth reifying” Normative
Who to involve	Customer	Many stakeholders
View on the future	The future of a business can be predicted	The future can and should be shaped
Similarities	Quick customer/stakeholder-involved learning Low cost, time, or resource method	

### Research Focus

Given the potential complementarities between effectuated and experimental approaches — combined with a lack of consensus in the literature regarding how the two can and should be reconciled — this paper offers a novel means of leveraging both logic by combining lean startup with effectuated thinking. Previous research has considered what tools or approaches might complement both effectuation and lean startup independently. For instance, Glen et al. [56, p. 662] propose design thinking as a “useful front end” process which can precede *either* lean startup *or* effectuated approaches to entrepreneurial action. Berglund et al. [54, p. 828] even juxtaposed experimentation and effectuation as distinct “ideal types.”

Yet, to our knowledge, there has been no concerted attempt to combine the two, particularly in a sustainability context. Furthermore, both effectuation and lean startup methodologies have been critiqued for failing to facilitate actual ideation processes [56]. By combining these two methodologies together and conducting a series of workshops (as detailed below), we aim to provide counterevidence to this claim.

In addition to providing these insights, a workshop process for circular business model experimentation is developed. To date, several tools have been developed to support sustainable and circular business model experimentation [27], a tool being a generic name for frameworks, models, concepts, or methods that codify knowledge and make it useful for researchers and practitioners to improve their decisions and actions [5, 57]. The business model canvas by Osterwalder and Pigneur [58] is a generic business model innovation tool used in other contexts [23]. Sustainability variants of the canvas have been developed such as the triple bottom line canvas by Joyce and Paquin [59], including the three layers of the triple bottom line (people, profit, planet), and the flourishing canvas by Upward and Jones [60]. Various workshop-based tools have been developed for sustainable business model innovation [32, 50]. Other tools include gamification [61]. According to a circular and sustainable business model review by Pieroni et al. [27], only 20% of the identified tools and methods were suited for the transforming stage of business model innovation, including activities such as experimenting, piloting, and implementing new business model concepts. Notably, at the time of initiating the first workshop (May 2016), few tools existed for circular business model innovation as the circular economy concept just started to gain popularity. The work by Pieroni et al. [27] shows that the earliest circular business model tools emerged from master theses (e.g., [62]), conferences (e.g., [63]), or from gray literature [64]. Pieroni et al. [27] point out that experimentation only started to emerge later as a theme recently (e.g., [65]).

Former research also suggested that few business and engineering tools for sustainability are effectively used in practice which is owed to the fact that those tools are not developed with the user in mind [66]. Hence, Bocken et al. [26] created a brief checklist for circular business tool development including various points such as the tool being circular economy specific, iteratively, and rigorously developed, and being used multiple times with the target group. This same research concluded that while a large number of tools for sustainable and circular business model innovation have been developed in the literature, only a small fraction of these satisfies three important design requirements: rigorous development (grounded in theory), validation from practice, and the presentation of a clear procedure for users. Hence, these points were taken into mind when developing a tool and process.

## Method

**Fig. 1:**
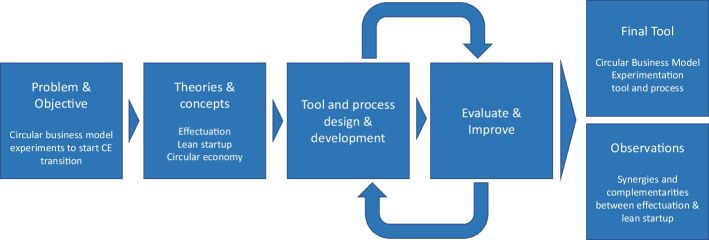
Research process. Building on [67, 68]. CE refers to circular economy

This research uses an action-oriented design science method [32, 67, 68]. When adopting such a method, initial theories lead to a certain design solution (in this case, a workshop process tool) that is used and tested in practice (the workshop being run with innovators), and subsequent observations iteratively lead to an improved process or tool [67]. See Fig. 1.

We started with a practical need and objective to support companies in their transition toward a circular business. This was addressed by circular business model experiments that are seen as a pathway to transitioning toward a circular business, or as an important process in emerging startups [35, 69]. The goal was to develop a tool to help innovators and entrepreneurs design and develop experiments for circular business model innovation. The intended user groups include entrepreneurs, innovation or R&D managers, strategists and business model innovators, consultants, and designers who want to innovate business models for a circular economy.

The theoretical starting point was the use of lean startup and effectual logic to be used in a workshop setting. In the workshops, the authors aimed to inspire others (innovators in companies, entrepreneurs, researchers) to innovate and experiment with the circular economy. While no known circular business model innovation or experimentation processes existed in peer-reviewed literature when the workshops started, as the circular economy phase was only starting to emerge in a business context [8], the authors drew on the knowledge of existing innovation methods suitable for low resource and time settings. This was deemed suitable as most companies involved had little resources and time involved for such innovation but were interested in making a start and being inspired to innovate for the circular economy. Notions from lean startup and effectuation were both seen as highly relevant in this context as they are suitable in an entrepreneurial context.

As the tool seeks to spur users to consider circular economy, initially, several emerging circular economy examples from industry were used to enrich the discussion. A tool to support circular economy experiment inspiration was introduced in the final workshop process, the circularity deck by Konietzko et al. [5]. This tool gives inspiration on the circular strategies (narrow, slow, close, regenerate resource loops) that could be adopted by companies and is embedded in the final version of the process.

A total of 10 workshops were conducted, which combined lean startup, effectuation, and circular economy thinking. The period covers the period of 2016–2022 during which the authors developed and iteratively tested an approach to support circular business model innovation and experimentation. Table 2 includes an overview of how the tool incorporates elements of rigid tool development, such as testing the tool with the user group.

**Table 2:** Tool criteria (criteria based on [26])

Tool criteria	How are these used in tool development
The tool is purpose-made	Focused on circular business model experimentation
The tool is rigorously developed—from literature and practice	Including effectual and lean startup logic, as well as circular economy literature, and tested in practice
The tool is iteratively developed and tested with potential users	Tested with the target audience
The final tool version has then been used multiple times by practitioners, and an evaluation of this process is done to assess tool use and usefulness	The final tool is tested 3 times and evaluated with a form and by the facilitators
The tool provides a transparent procedure and guidance	A structured, stepwise process used in the virtual tool
Circular economy or broader sustainability objectives and impact are firmly integrated	The circularity deck is used to incorporate circularity concerns
Simple and not too time-consuming	The final version is created so it can be completed in a 1.5-h virtual workshop
Inspires or triggers change	The goal of the tool is to inspire circular inspiration
Adaptable to different (business) contexts	Developed for both startups and existing business

Table 3 provides an overview of the conducted workshops. Workshops 1–7 consisted of similar types of workshops where the process was iteratively improved based on the experience of using the tool in practice. The final tool was used in workshops 8–10. The main change in the final version of the tool was that both lean startup and effectuation principles were briefly explained at the start of these workshops. Furthermore, effectuation principles were added specifically to set the scene and refine experiments. While in other workshops, lean and effectual principles were already used, they became more prominent in the final workshop setting.

**Table 3:** Workshops conducted for this study

#	Workshop	Place and time	Lean startup elements	Effectual elements	Circular economy elements
Iterations					
1	Workshop with businesses of different sizes (~ 50 participants)	Finland, May 2016	Prompt about what lean startup is; hypothesis development, testing measures, and success criteria to create new circular business ideas, in a low-cost and iterative way	Prompts about who they are, what they know, what they have	Industry examples, prioritize according to impact (vs. feasibility), sustainable business model canvas
2	Workshop with PhD researchers in circular economy (~ 20 participants)	Denmark, November 2017	Prompt about what lean startup is; hypothesis development, testing measures, and success criteria to create new circular business ideas, in a low-cost and iterative way	Prompts about low cost and using available means and resources	Industry examples, prioritize according to impact (vs. feasibility, sustainable business model canvas
3	Workshop with PhD researchers in circular economy (~ 20 participants)	Denmark, November 2018	Prompt about what lean startup is; hypothesis development, testing measures, and success criteria to create new circular business ideas, in a low-cost and iterative way	Prompts about low cost and using available means and resources	Industry examples, prioritize according to impact (vs. feasibility), sustainable business model canvas
4	Workshop with 40 international business managers	UK, April 2019	Prompts about iteratively improving the value proposition; deliberate learning through value proposition iterations; low-cost testing	Working with stakeholders more effectively and efficiently to collaboratively address societal issues	Sustainable business model canvas, value mapping tool, sustainability idea cards; prioritize according to impact (vs. feasibility)
5	Workshop with ~ 25 European entrepreneurs	Sweden, October 2019	Prompts about iteratively improving the value proposition	Own perspective, ecosystems and stakeholder perspective, how to be the “pilot in the plane” (influence the ecosystem)	Circularity card deck with industry examples; conscious mapping of synergies between customer and circular economy proposition
6	Workshop with 5 business participants of the same company *(sustainable scale-up)*	The Netherlands, December 2020	Hypothesis development, testing measures, and success criteria to create new circular business ideas, in a low-cost and iterative way	Questions: Who are you, and what is your role? What drives you? What data do they have as a starting point?	Industry examples; prioritize according to impact (vs. feasibility)
7	Workshop with 3 business participants of the same company *(sustainable scale-up)*	Virtual, June 2021	Hypothesis development, testing measures, and success criteria to create new circular business ideas, in a low-cost and iterative way	Prompts about low cost and using available means and resources	Industry examples; circularity card deck; ask to prioritize most impactful examples
Final tool					
8	Workshop with 18 business participants *(innovators in circular economy, globally*)	Virtual, February 2022	Explain the principles of lean startup. Hypothesis development, testing measures, and success criteria to create new circular business ideas, in a low-cost and iterative way	Explain principles of effectuation explicitly. Added: (1) Who they are and how they contribute to shaping the future; (2) What they can accept to lose, (3) Whom they know (4) What they can influence with whom (5)	Industry examples; circularity card deck
9	Workshop with 13 business participants *(innovators and consultants in circular economy, globally*)	Virtual, June 2022	Same as above	Same as above	Same as above
10	Workshop with 16 business participants *(innovators in circular economy, globally*)	Virtual, July 2022	Same as above	Same as above	Same as above

This final version of the workshop tool (Appendix 1) was used three times in a virtual workshop setting. For workshops 8–10 where the final tool was used, an evaluation form, using Google Forms, was used to assess the usefulness of the tool (see Appendix 2 for the main questions). This was supplemented by the experiences of the facilitators of the tool, as discussed after each workshop. Based on these, the authors developed propositions guiding future research in relation to effectuation and lean startup for circular business model innovation processes and specifically the development of experiments. See also Fig. 1.

The final workshop process (“Final Workshop Process”) as well as the findings on the compatibility of effectuation and lean startup (“Evaluation of Final Workshop Process”) are discussed next.

## Results

In the “Results” section, we first discuss the development of the final workshop process (“Final Workshop Process”), followed by the quantitative and qualitative evaluation of the workshop (“Evaluation of Final Workshop Process”).

### Final Workshop Process

The workshop process was iteratively developed. In workshops 1–7, a similar process was used, building on lean startup principles as a starting point, but using some effectual prompts in addition. The main changes after these sessions were the addition of both an explicit (but brief) explanation of lean startup and effectuation principles and the inclusion of all effectuation principles. The workshop was also created for virtual participation on the online collaborative platform Miro. In this way, the workshop could be conducted with bigger groups virtually, and a larger audience of innovators could be reached.

The final workshop process that fits within a 1.5-h format was used three times with circular economy innovators (sessions 8–10 of Table 3). The final process looks as follows (see also Appendix 1 for the visual tool):

- Introduction in plenary form (10 min):
  Here, the aim of inspiring participants to develop circular business model experiments is explained. The concepts of lean startup and effectuation are briefly explained.
- Three breakout sessions (in total 70 min):
  Breakout session one (10 min) is about the innovators’ starting point: What is their circularity challenge? For example, a company may be considering a shampoo refill station, or a clothing exchange platform, electric car leasing, etc. This is the first time that they join a virtual breakout group (food, mobility, etc.). Effectual questions are asked around what they find important (introspective part), how they want to shape the future (pilot in the plane) and what trends and uncertainties influence their business, and how negatives can be turned into positives (lemonade principle).
- Breakout session two is a “circularity brainstorm” to refine initial ideas (20 min). Prominent examples from the circularity card deck including strategies to close, slow, narrow, and regenerate loops [5] are used to get inspired to develop and refine ideas for circular business models
- Breakout session three is about experiment design (40 min). Innovators are asked to think about a hypothesis, test, and measures of success. They get inspired to form an initial experiment based on what they accept to lose (affordable loss), what and who they know (bird in hand and crazy quilt), how they can leverage uncertainty and unexpected opportunities (lemonade principle), and who they can influence (pilot in the plane). If there is time left, they can define measures and success criteria. Hence, effectual principles are used to inspire lean startup-type experiment design.
- Closure (10 min):

Any final reflections, sharing of the results, and feedback form.

### Evaluation of Final Workshop Process

Workshops 8–10, where the final process was used, were evaluated using the same feedback form (Appendix 2). Participants were asked to evaluate how easy the workshop was to follow and how useful it was. Overall, the scores were very positive, where the workshop was seen as easy to follow (4.11 on average) and useful (4.35 on average), measured on a 5-point Likert scale where 1 is “not very” and 5 is “very much” (Table 4).

**Table 4:** Results from the evaluation

	Workshop 1	Workshop 2	Workshop 3	Overall assessment
How easy was the workshop to follow? *(mean and standard deviation)*	4.15 (0.69)	4 (0.63)	4.17 (0.49)	**4.11**
How useful was the workshop for you? *(mean and standard deviation)*	4.23 (0.83)	4.5 (0.84)	4.33 (0.53)	**4.35**
Number of respondents and participants	13 (18 participants)	6 (13 participants)	7 (16 participants)	

In addition, participants were asked for key takeaways and ways to improve the workshop. Table 5 contains the qualitative assessment based on the free text spaces filled in by participants. In general, the process lived up to its expectation of providing a starting point for quick iterative circular business model experimentation. However, there were some suggestions to improve the explanation, selection of participants, process, and preparation (Table 5). However, they did not change the main format of the tool.

**Table 5:** Qualitative assessment

Key takeaways	Suggestions for improvement	Actions
Provided useful new methods (e.g., “the effectuation theory and experiment design,” “clear frameworks.” “circularity deck”)	Explanation: - Additional intro or guidance - Written step-by-step process	- Allocate sufficient time for explanation - The final tool may include additional explanation text boxes to make it self-explanatory
Collaboration is fun and helpful (“co-creation is ultra great,” “connection and group work,” “always inspiring to hear other peoples stories”)	Participants: - Do this with a multidisciplinary team - More people from the same sector	- Organize future sessions with specific teams within companies and/or take more time to select participants
Lean aspects were appreciated (“use less resources to implement a circular business model,” “simplicity and rapidity to develop ideas,” “quick inspiration”)	Process: - More time/a little more time for the process - Series of workshops for the same team	- Extend the tool to a 2–2.5-h session when the setting allows for it - Incorporate the tool into long-term intrapreneurial innovation processes
Circularity aspects were helpful (“the circular deck,” “circularity deck and tools,” “circular deck and process”)	Preparation: - Specific circularity challenges to prepare in advance	- When organizing sessions, pre-select challenges or ask companies to choose specific ones to deal with in the group

## Discussion

Previous circular economy studies have mapped existing tools and methods (e.g., [27]). Researchers have also noted the benefits of effectual thinking for the circular economy [70] or developed tools and methods that incorporated such thinking [50]. Others have investigated the value of lean startup for circular business experiments (e.g., [24]). This study makes two specific contributions: (1) the development of a workshop process that embeds the logic of both concepts and (2) a deeper understanding of the synergies and complementarities between both methods. In the following, we first discuss the contributions in more detail (“The Synergistic Use of Effectuation and Lean Startup”), followed by suggestions for future research and practice and the limitations (“Future Research and Practice”).

### The Synergistic Use of Effectuation and Lean Startup

This research found that the logic of lean startup and effectuation can be bridged successfully for the circular business model innovation process. In contrast to the argument about the incompatibility between more causal and effectual reasoning [51], we rather suggest that the more causal lean startup type of approach prominent in mainstream business (e.g., [22]) can be enriched by effectuation principles if used in the right way and vice versa. Former studies already stated the value of effectual reasoning for innovativeness [43] and lead to the development of more responsible business practices [44]. Vice versa, lean startup type of logic can support the development of (sustainable) business model innovations in established businesses, confirming earlier research by Bocken and Snihur [23] and Weissbrod and Bocken [24].

In the present study, we found that, first, the starting point of the workshop was helpful to understand how innovators can be impactful in the grand circular economy transition. We found that the effectual questions – what innovators find important, how they want to shape the future, what trends and uncertainties influence their business, and how negatives can be turned into positives – provided them with a focus on where they can be influential in the grand circular economy transition. In the workshops, broad ideas became much more focused.

This leads us to the following proposition:

#### Proposition 1

Effectual questions about the innovators’ starting point – what they find important, how they want to shape the future, what trends and uncertainties influence their business, and how negatives can be turned into positives – can help them focus on where they can specifically be influential in the grand circular economy transition.

When developing actual experiments, many of the effectuation principles (e.g., lemonade principle, crazy quilt [25]) can provide practical guidance on how to set up practical, low-cost, and resource experiments prominent in lean startup [21]. We found that the effectual guidance helped innovators develop circular business model experiments more easily. Focusing on building on who and what is available and making the most of adverse situations is particularly useful in a volatile, uncertain, complex, and ambiguous (VUCA) world [42]. For instance, the COVID-19 pandemic forced businesses like restaurants to pivot their business models quickly [71]. Effectuation principles, like building on who and what is available, making the most of adverse situations, were found to provide useful input to circular business model experiment development.

Moreover, thinking about whom you share a circular challenge with or who could support your circular economy challenge can enrich circular business model experiments. Finding collaborators to join your challenge is also common in circular business practice [69]. For example, the Net-Works program is a collaboration between the Zoological Society of London, carpet manufacturer interface, and nylon manufacturer Aquafil, who together work on a solution to create new carpets out of (formerly) discarded fishing nets and avoid further disposal of fishing nets in the ocean [69]. We found that effectual logic can support the development of lean experiments also to solve circular economy challenges collaboratively. This was especially evident in workshops 8–10, where participants joined the workshop with overlapping interests and circularity challenges but often distinct networks, skill sets, and access to resources. The “crazy quilt” and “pilot-in-the-plane” aspects of effectuation thus became especially relevant, suggesting the importance of reaching outside one’s existing organization and increasing multi-stakeholder collaboration when attempting to develop circular business models.

This leads us to the following proposition:

#### Proposition 2

Using effectual logic, focused on building on who and what is available, making the most of difficult situations, as well as working with those stakeholders that can jointly exercise influence on the specific circular economy challenge, could support and enrich the lean logic needed for circular business model experiments.

Conversely, the lean startup focuses on the customer and competitive positioning [20, 21], and the structure of cycles of experiments might add practical value to the effectual approach. In the workshop, the structured approach of ideas, hypotheses, tests, measures, and success criteria helped bring focus to the broader circular economy discussions. It helped innovators formulate more precise circular business model experiments for problems and challenges that started as broad wicked issues such as “plastic soup” and “textile waste.” While thinking in effectuated terms can help entrepreneurs leverage contingencies and “co-create hypotheses worth reifying” [51, p. 1], it is through clarifying, testing, learning from, and iterating upon these hypotheses that new circular business models can emerge in practice.

#### Proposition 3

Effectual entrepreneurs and innovators seeking to tackle wicked issues prominent in the circular economy transition might benefit from the structure provided by lean startup, as this practical guidance can help them to develop concrete circular business model experiments to start addressing these grand challenges.

### Future Research and Practice

The space in which businesses operate has become riskier, but also more volatile, uncertain, complex, and ambiguous [42]. The effects of a warming climate are already noticeable, biodiversity is in decline [72], and access to resources is an increasing business risk [73]. The circular economy is positioned as a paradigm to address not only urgent action to climate change [74], waste, and resource issues but also the criticality of raw materials and future competitiveness [75, 76]. Yet, existing companies typically still have a long way to go in their transition to a circular model, and while there are many emerging circular startups, many have failed to achieve scale [19, 38]. Hence, experimentation has become so important to trial new business models in practice and challenge dominant linear models [33].

First, in this research, we found that effectual logic can enrich lean startup type of experiment development. Effectual questions about what is important and how one can influence and shape the future (“pilot in the plane”) under potentially adverse conditions (“lemonade principle”) can help shape the innovators’ focus within a circular economy. Furthermore, seeking out which stakeholders to experiment with (“crazy quilt”) can help scale-up experiments more easily. While we did not test this explicitly, we suggest that the inclusion of stakeholders in innovation processes common in effectual logic can enrich the development of more systematic solutions needed for circular business model innovation. This confirms earlier research that suggests that early stakeholder involvement is needed for the sustainable and circular business model innovation process [12, 13, 50, 77].

Second, effectual entrepreneurs might benefit from the structure and customer focus that lean startup type of logic Blank [13] provides. The focus on testing early variations of business models with prospective customers as well as the positioning of the value proposition compared to the competition in lean startup provides a practical angle to enrich effectual logic.

Third, there are many synergies between the logic that can be leveraged. The combination of quick customer/stakeholder-involved learning and the low cost, time, or resource method of both effectuation and lean startup, combined with the explicit focus on stakeholder-involved problem solving common in effectual logic, can help inspire solutions to the wicked societal problems such as the circular economy transition. Table 6 compares both approaches and makes suggestions for synergies. The similarities and complementarities could provide useful starting points for future work.

**Table 6:** The lean startup vs. the effectual approach

	Lean startup	Effectuation
Premises	Iterative build-measure-learn cycles	Start with available means: who you are, what you know, who you know
Focus	Expected return	Affordable loss
Competition vs. collaboration	Understanding competitive positioning	Forging strategic partnerships
Method	Scientific method	Entrepreneurial method
Approach	Scientific approach (as if) Knowledge and prediction Test hypotheses, e.g., A-B split testing “Value-neutral”	Effectual approach (even if) Leveraging contingencies Co-create hypotheses “worth verifying” Normative
Who to involve	Customer	Many stakeholders
View on the future	The future of a business can be predicted	The future can and should be shaped
Similarities	Quick customer/stakeholder-involved learning Low cost, time, or resource method	
Complementarities/synergies	Adds a structured approach to innovation* Focuses on competition and deeply understanding the customer Emphasizes early action and iteration	- Geared toward resolving wicked societal problems - Gives focus on what circular economy challenge to solve through effectual questions* - Supports identification of low-cost/resource experiments by emphasizing what/who is known and available* - Emphasizes inclusion of stakeholders that can influence the outcome in addition to customers*

Complementarities based on findings. Building on [21, 25, 47, 51]. An asterisk (*) denotes that this was an explicit finding from this study.

Future research may also focus on principles that were less prominent in this study, such as a broad interpretation of the effectuation principle of “affordable loss” [25]. Within the time and contextual constraints imposed by our test workshops, the principle of “affordable loss” received less focus and attention. In practitioner contexts, however, a more explicit focus on this principle as part of the circular experimentation process could add considerable value. First, as suggested by Coffay et al. [55], the traditional understanding of affordable loss can be extended to include not only just financial considerations (e.g., how much can we afford to lose if we invest in this idea?) but also environmental ones (e.g., where do we draw the line on emissions or nonrenewable resource consumption?). Furthermore, paying attention to affordable loss in effectuation terms helps to bridge the gap between sustainable and circular business model innovation practice on the one hand, and more “conventional” business model innovation (BMI) practice on the other, where building business model innovation funnels and portfolios imply a willingness to make limited investments in new ideas with the understanding that many of them will lead to short-term losses [78]. In much the same way that successful venture capital investment is predicated upon a large number of failed ventures and a small number of successful ones — with accompanying proportional losses and returns — success in business model innovation in corporate contexts typically requires the willingness to make rational “bets” on intrapreneurial projects, many of which will fail, but some of which will lead to new revenue streams. Operating with affordable loss (rather than a myopic focus on return on investment) as a starting principle is therefore important not just in “conventional” BMI contexts, but in circular and sustainable business model experimentation processes as well, where big wins take the form of both future revenue streams and substantial improvements in sustainability and circularity metrics.

Finally, the Circular Experimentation Workbench process ends with the development of specific experiments. In addition, the business model canvas [58] or lean canvas [21], or sustainability (e.g., [59, 60, 65] or a circular economy variety of the business model canvas (e.g., [77, 79]) could supplement the workshop. It could either be used as a follow-up or as part of the ideation phase to explore and map circular business models in more detail. Observation of workshop participants engaged in these aspects of the Circular Experimentation Workbench indicated that mapping the business model onto an extant canvas could contribute to a clearer conceptualization of the emergent business idea, as well as facilitate discussion between participants. It could also improve the ability of participants to extract implicit assumptions from the business model idea, thereby facilitating the formulation of testable hypotheses.

### Limitations

This research also has some limitations related to the workshop format, action-oriented research, and the use of a virtual setting for research.

First, the use of workshop formats to simultaneously develop a tool and gather insight data is still rather new and untested. The sustainability tool development process is certainly not new and has been common for over two decades in design, engineering, and business studies [66]. Yet, the rigidity of tool development in research and practice is still insufficient, leading to many tools remaining unused in practice [26]. We have sought to overcome this limitation by rigorous development (grounded in theory), validation from practice, and the presentation of a clear procedure for users.

Second, while action-oriented research methods are gaining ground and are much needed in sustainability and circularity research to accelerate the transition, they might lead to role conflicts [80]. Being part of the action may have led to viewing the results more positively. Furthermore, experienced facilitators may also influence the outcomes [50, 81]. While the feedback on the overall process was positive (see Table 4), on average, 54% of workshop participants filled out the online survey. This could have influenced the outcome as not all participants gave written feedback. Ultimately, the sustainability transition might require a different role for academia in relation to business, with researchers engaging in more participatory forms of research and innovation — a transition that may already be on its way [80, 82]. Hence, it would be recommended to further develop action-based methods for the circular economy transition and develop appropriate evaluation methods.

Third, the final tool was only used in a virtual setting. While earlier versions of the tool were used with businesses in a face-to-face setting, it would be worthwhile to test the final version again with a face-to-face audience to better determine its value.

## Conclusions

Given the urgency to address climate change and its negative impacts on biodiversity and people, as well as exacerbating waste and resource issues, it is becoming increasingly pressing to put the circular economy paradigm into practice. Circular business models such as second-hand offerings or rental platforms allowing for reuse and recycling provide a way to holistically address circular economy issues in a business context. It is important that established companies, who take up a large part of the innovation landscape, start experimenting with circular business models to challenge their dominant linear business models. To date, however, there are limited tools which companies can leverage for this type of experimentation.

In this paper, we build on lean startup, effectuation, and circular economy thinking to address this challenge. Lean startup and effectuation have been tried and tested in a startup context, but their value in a corporate or established business context is only starting to be explored (e.g., [24, 43, 44]. We investigated the following research question: To what extent can lean startup and effectual thinking be combined to support the circular business model innovation process? Using an action-oriented design science method, we conducted 10 workshops where we combined lean startup, effectuation, and circular economy thinking. This led to two key outcomes: (1) an evaluation of how lean startup and effectuation principles may be combined, and (2) a final tool, the Circular Experimentation Workbench.

First, this study contributes to research by a novel integration of lean startup, effectuation, and circular economy thinking by demonstrating its potential for combined usage in practice. It was found that lean startup and effectuation can be used in low resource and time settings. Effectual questions can support the focused development of experiments in the broad area of circular economy. Moreover, effectual logic – e.g., working with familiar stakeholders and making the most of what is available – can also enrich the lean logic needed for experimentation. Finally, while effectual entrepreneurs might seek to tackle wicked societal challenges, the lean startup can provide a structured approach to innovation.

Second, the novel Circular Experimentation Workbench was developed, so-called, as it integrates tools and approaches from different fields: lean startup [21], effectuation [25], and the circularity card deck [5]. By inspiring new circular experiments in different contexts, this tool was found to support the development of circular business models: innovators using the tool evaluated it as useful and easy to follow, commenting specifically on the usefulness of the process, principles, and circular economy inspiration.

As a contribution to practitioners, through this work, we aim to motivate those working in businesses to start experimenting with circular business models to challenge the still largely linear, unsustainable business models present omnipresent across industries [18, 83]. For policymakers, we see much value in the further development of the Circular Economy action plan as part of the European Green Deal. We encourage the nurturing of experimentation spaces for businesses and industries, and transdisciplinary partnerships. In addition, clear pathways are needed for business through sector-specific circular economy policies (necessitating repair, availability of spare parts, product longevity, etc.). The creation of such pathways could pave the way for similar models and levels of adoption in other parts of the world [84].

Future research could build on the complementary perspectives of lean startup and effectuation to help accelerate the circular economy transition through not only encouraging experimentation but also scaling up initiatives. Methodologically, action-based methods can be useful to simultaneously advance research and practice for pressing issues such as the climate crisis.

## Supplementary Information

Below is the link to the electronic supplementary material.

Supplementary file1 (DOCX 511 KB)

## Data Availability

Data on specific workshop outcomes or individuals and their organizations are not available. The main outcome is the workshop process which is made available. The Circular Experimentation Workbench workshop template can be accessed freely here: https://miro.com/miroverse/circular-experimentation-workbench/.
