# Supplementary material for: The Circular Experimentation Workbench – a Lean and Effectual Process
Source: Circ Econ Sustain. 2022 Dec 7;3(3):1361–83. doi: 10.1007/s43615-022-00239-w (PMC9734756; doi:10.1007/s43615-022-00239-w)
Supplement: Supplementary file 1 — Supplementary file1 (DOCX 511 KB) [file 43615_2022_239_MOESM1_ESM.docx]

**Appendices**

**Appendix A.** Circular Experimentation Workbench process


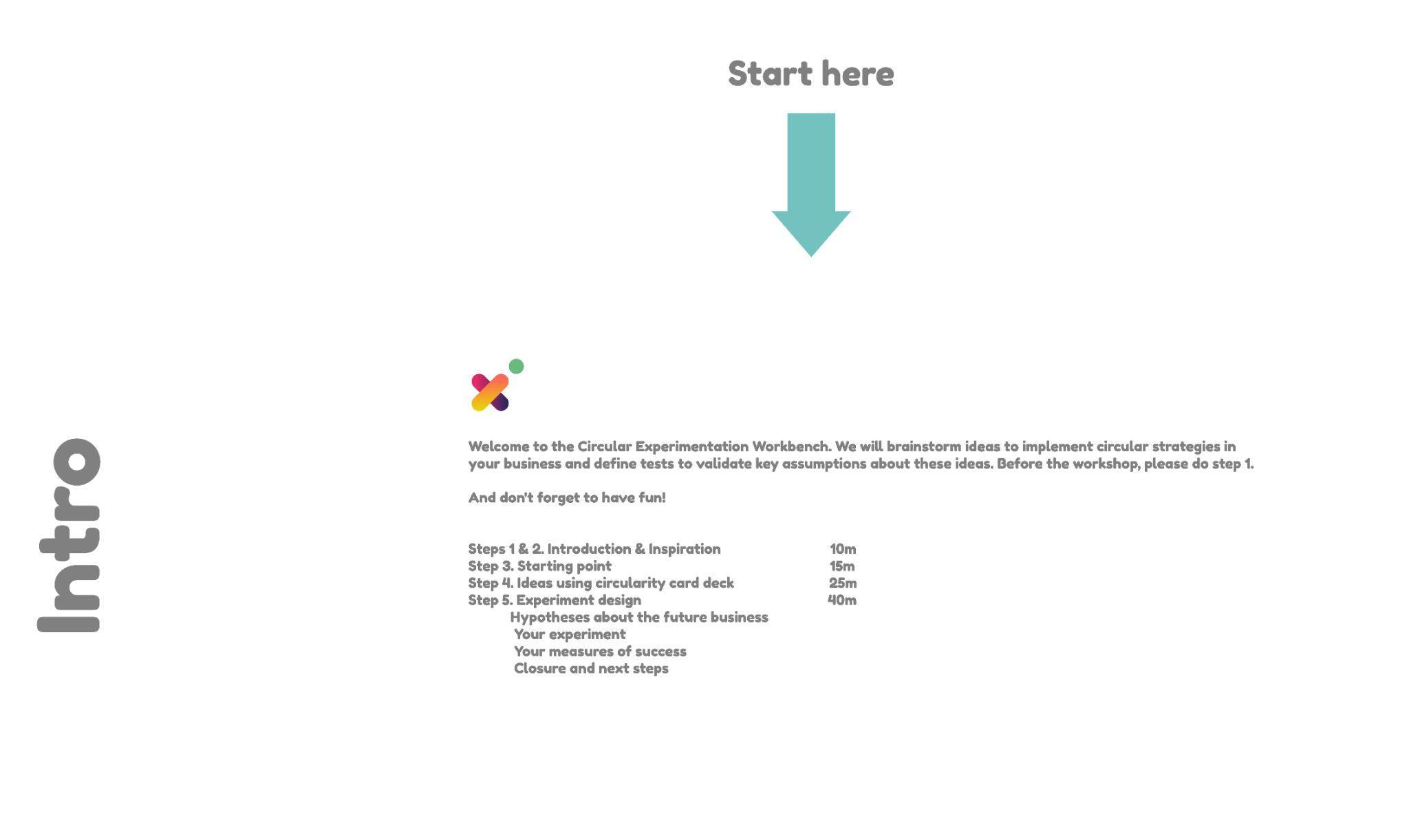


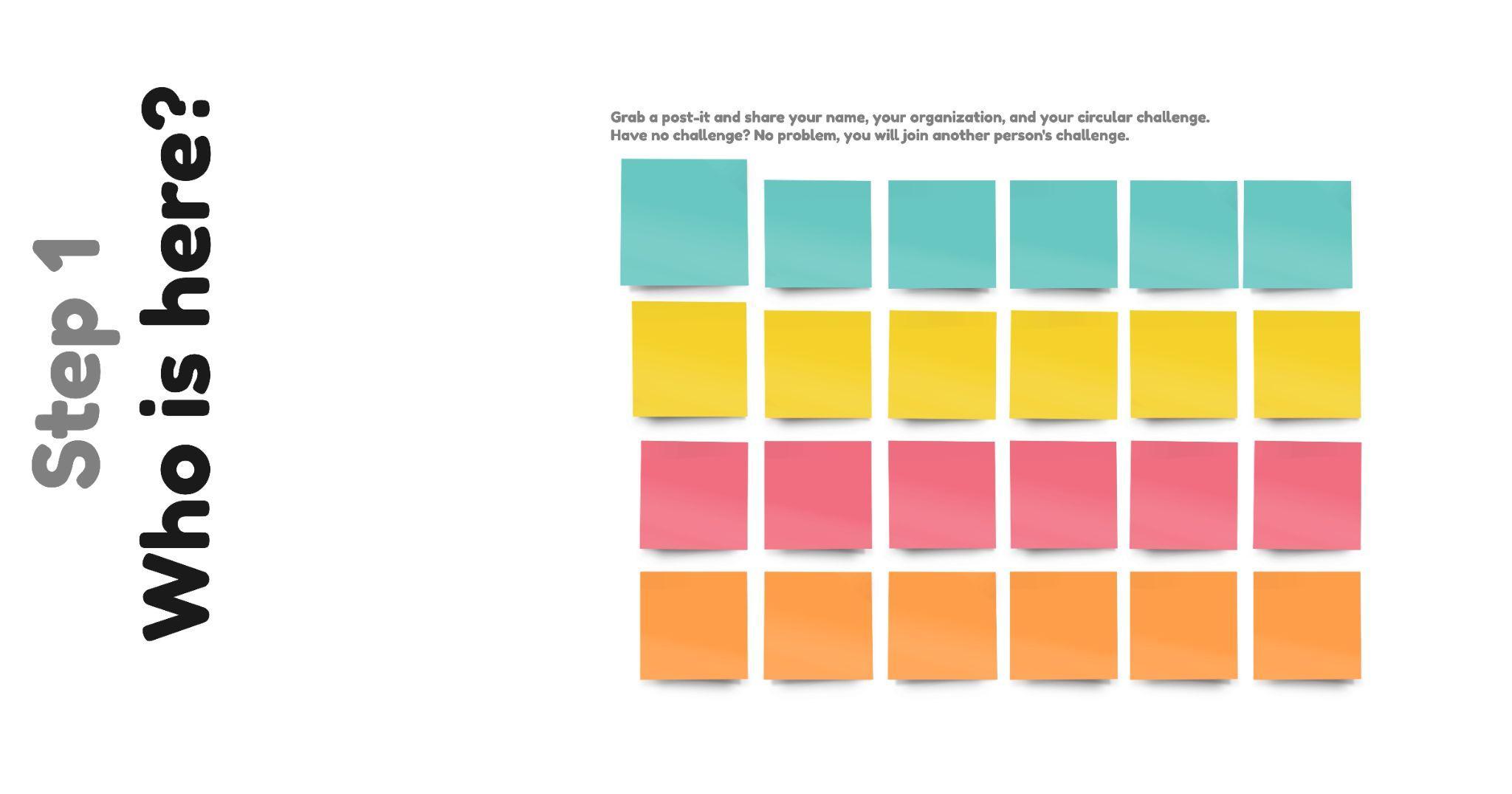


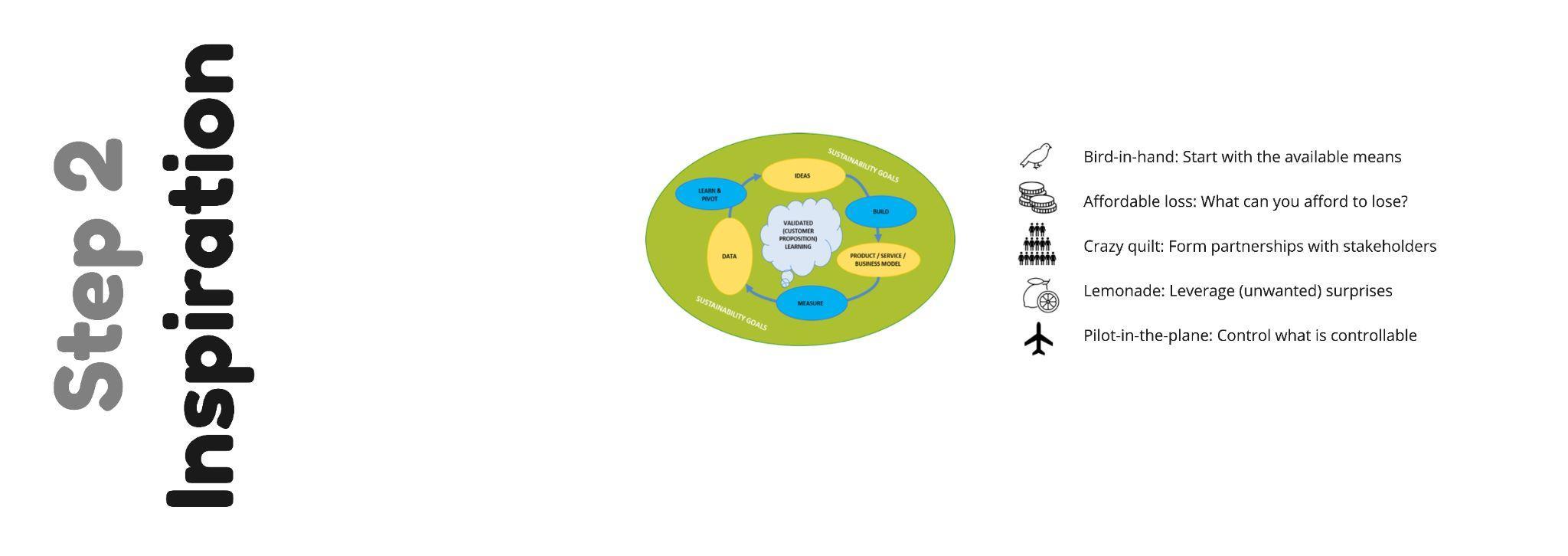


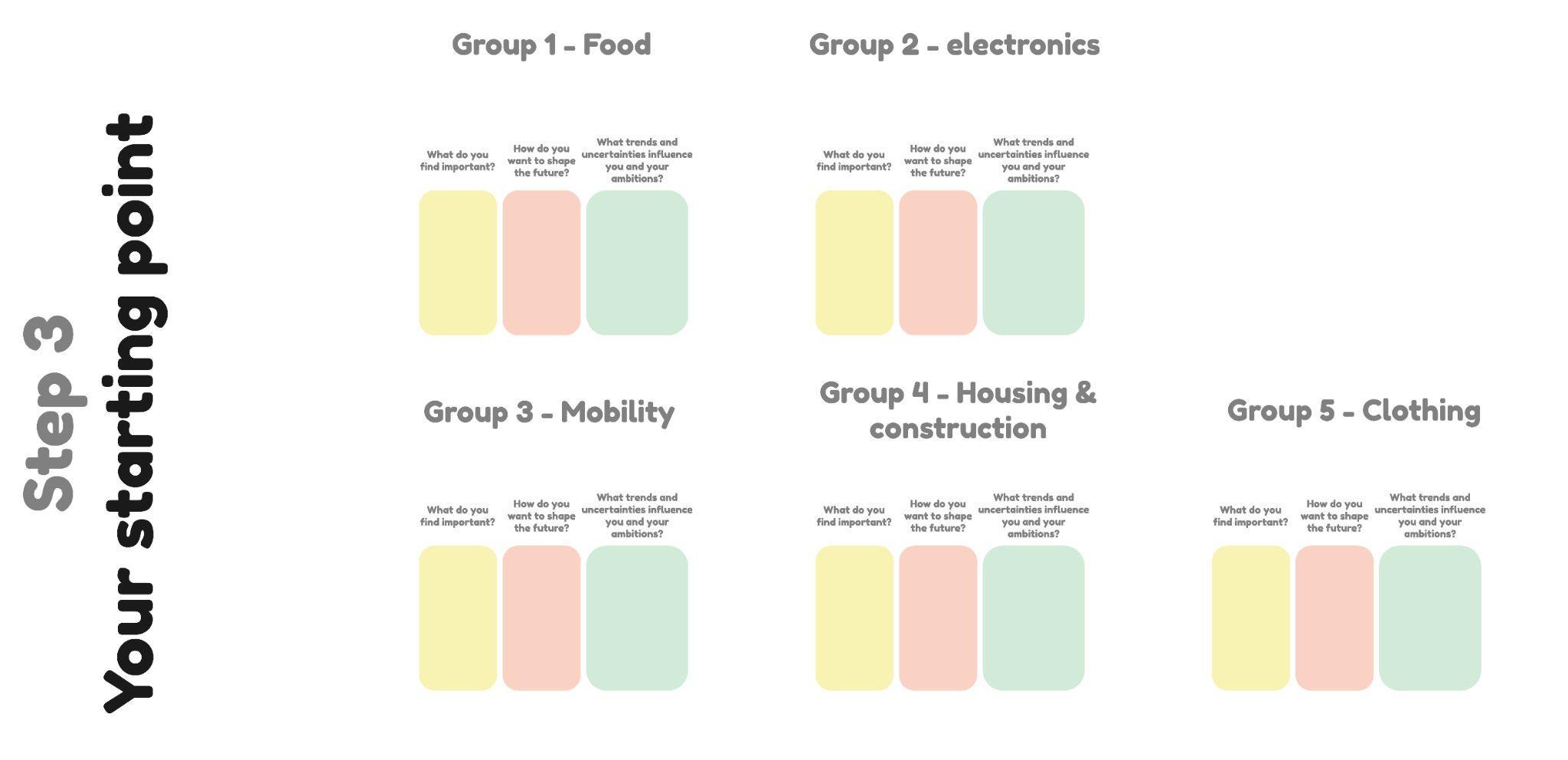


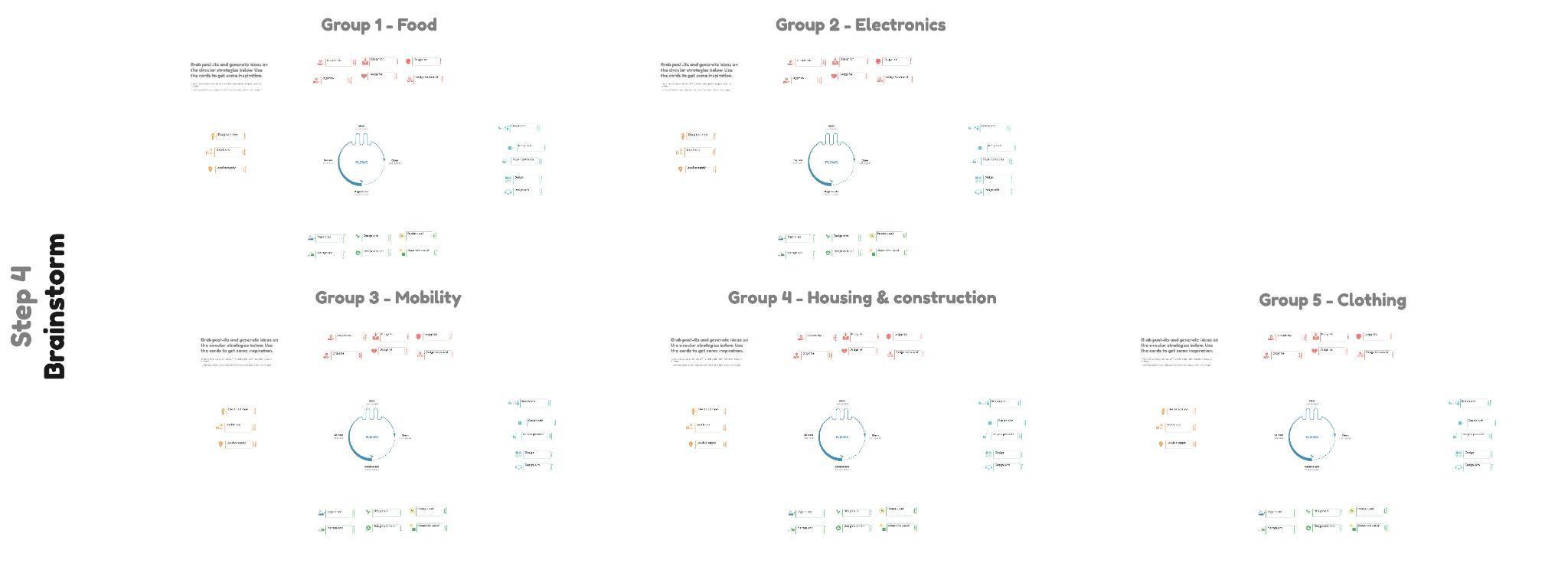


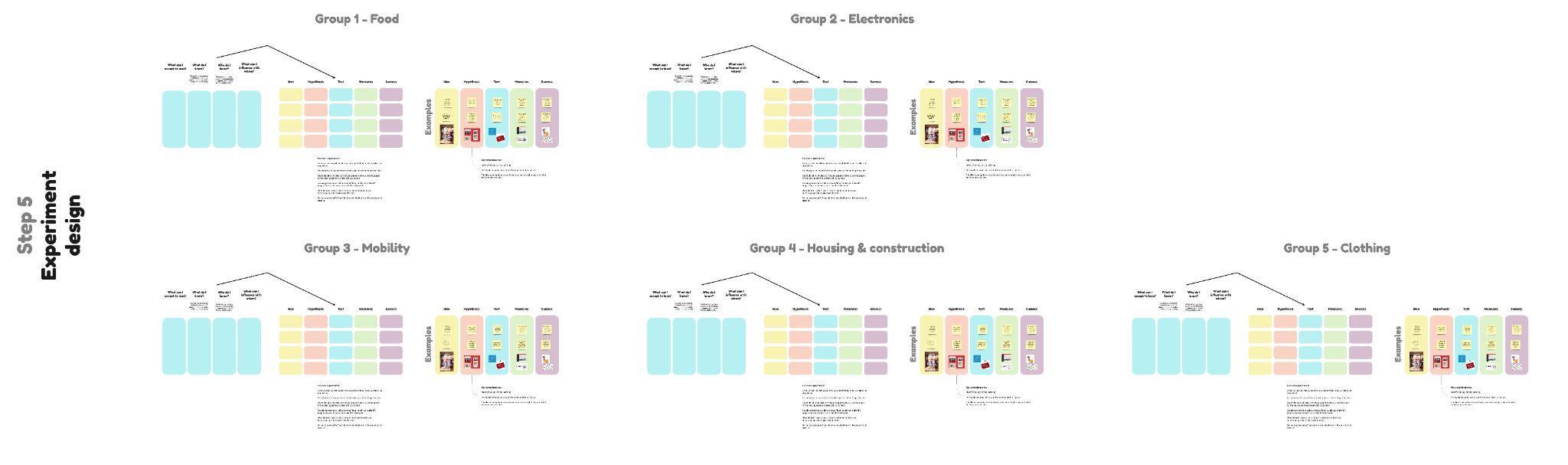


**Appendix B.** Feedback form - main questions

- How easy was the workshop to follow?

1 2 3 4 5

0 0 0 0 0

Not very Very much

- How useful was the workshop for you?

1 2 3 4 5

0 0 0 0 0

Not very Very much

- What were the key takeaways from this workshop?

*…. Your answer ….*

How can we improve the workshop?

*…. Your answer ….*
